# Repotrectinib (TPX-0005), effectively reduces growth of ALK driven neuroblastoma cells

**DOI:** 10.1038/s41598-019-55060-7

**Published:** 2019-12-18

**Authors:** Diana Cervantes-Madrid, Joanna Szydzik, Dan Emil Lind, Marcus Borenäs, Mats Bemark, Jean Cui, Ruth Helen Palmer, Bengt Hallberg

**Affiliations:** 1 Department of Medical Biochemistry and Cell Biology, Institute of Biomedicine, Sahlgrenska Academy, University of Gothenburg, SE-405 30 Gothenburg, Sweden; 2 Mucosal Immunobiology and Vaccine Center (MIVAC), Department of Microbiology and Immunology, Institute of Biomedicine, University of Gothenburg, SE-405 30 Gothenburg, Sweden; 3 Turning Point Therapeutics, Inc. 10628 Science Center Drive, Suite 200, San Diego, California 92121 United States

**Keywords:** Drug development, Molecular medicine

## Abstract

Neuroblastoma is the most commonly diagnosed extracranial tumor in the first year of life. Approximately 9% of neuroblastoma patients present germline or somatic aberrations in the gene encoding for anaplastic lymphoma kinase (ALK). This increases in high-risk neuroblastomas, which have a 14% frequency of *ALK* aberrations at the time of diagnosis and show increasing numbers at relapse. Abrogating ALK activity with kinase inhibitors is employed as clinical therapy in malignancies such as non-small cell lung cancer and has shown good results in pediatric inflammatory myofibroblastic tumors and anaplastic large cell lymphomas. A phase I clinical trial of the first generation ALK inhibitor, crizotinib, in neuroblastoma patients showed modest results and suggested that further investigation was needed. Continuous development of ALK inhibitors has resulted in the third generation inhibitor repotrectinib (TPX-0005), which targets the active kinase conformations of ALK, ROS1 and TRK receptors. In the present study we investigated the effects of repotrectinib in a neuroblastoma setting *in vitro* and *in vivo*. Neuroblastoma cell lines were treated with repotrectinib to investigate inhibition of ALK and to determine its effect on proliferation. PC12 cells transfected with different ALK mutant variants were used to study the efficacy of repotrectinib to block ALK activation/signaling. The *in vivo* effect of repotrectinib was also analyzed in a neuroblastoma xenograft model. Our results show that repotrectinib is capable of inhibiting signaling activity of a range of ALK mutant variants found in neuroblastoma patients and importantly it exhibits strong antitumor effects in a xenograft model of neuroblastoma.

## Introduction

Neuroblastoma is an embryonal tumor of the sympathetic nervous system which is responsible for approximately 15% of all pediatric cancer deaths^1–3^. The primary tumor arises from the sympathetic nervous system and often in the vicinity of the adrenal glands^1–3^. Despite difficulties in predicting the outcome of neuroblastoma patients the International Neuroblastoma Risk Group (INRG) task force has established a stratification system. Neuroblastoma patients are grouped as having low, intermediate and high-risk disease depending on different prognostic factors i.e. age at time of diagnosis, clinical stage, tumor histology and tumor differentiation, chromosome 11q status, MYCN amplification and DNA ploidy^4,5^. Approximately 50% of newly diagnosed patients are classified as high-risk neuroblastoma, the 5 year survival rate for these patients is 40–50%^2,6,7^.

Alterations in the *ALK* gene are found in both familial and sporadic neuroblastoma cases, and at a higher frequency in the relapsed patient population^6,8,9^. ALK is a receptor tyrosine kinase (RTK) activated by the ALKAL ligands^10–16^. In vertebrates, ALK is expressed in the central and peripheral nervous system^12,14,17^. In mice ALK is not critically required during development although behavioral phenotypes and hormonal disturbances have been reported in knock out mice^18–21^. Although numerous mutations in *ALK* have been identified, three “hot spots” in the ALK kinase domain at residues F1174, F1245 and R1275 account for the majority of ALK aberrations in neuroblastoma patients^6^. These mutations facilitate ALK activation resulting in constitutive downstream signaling^22,23^.

Numerous ALK inhibitors have been developed, such as crizotinib, ceritinib, alectinib and brigatinib, and are used clinically for the treatment of patients with ALK-fusion positive tumors such as EML4-ALK positive non-small cell lung cancer (NSCLC)^24,25^. The initial crizotinib clinical trial in ALK positive pediatric cancers showed strong anti-tumor activity in patients harboring ALK fusions in inflammatory myofibroblastic tumors (IMTs) and anaplastic large cell lymphomas (ALCLs), but less impressive results in neuroblastoma patients, which express mutated variants of full-length ALK^26^. A recently presented follow-up study reported robust and sustained clinical responses to crizotinib therapy in pediatric patients with ALCL and IMT, stressing the importance of abrogating ALK kinase activity in these diseases^27^. In adult populations, despite the initial anti-tumor effect of ALK inhibitors, resistance appears often in the form of mutations in the ALK kinase domain or by-pass mechanisms, limiting clinical efficacy^28,29^, and highlighting the importance of the development of new ALK inhibition regimes that are better able to overcome relapsed ALK positive tumor growth.

Recently a new ALK inhibitor, repotrectinib, was developed^30^. This compound has a compact three-dimensional macrocyclic structure that allows it to bind within the ATP binding pocket of different kinases, including ALK, ROS1 and pan-TRK to avoid steric hindrance from the mutations of the kinase solvent front residues^30,31^. The high affinity of repotrectinib towards the adenine-binding site of ATP allows it to block both wild type and various mutant ALK activities. It has been shown that repotrectinib potently inhibits ALK as well as the related RTKs, ROS1 and TRKA-C^32^. Repotrectinib is currently under investigation in a phase 1/2 multi-center, first-in-human study to define safety, tolerability, pharmacokinetics and anti-tumor activity in patients with advanced solid tumors harboring ALK, ROS1, or NTRK1-3 rearrangements (TRIDENT-1, clinicaltrials.com). Preliminary results indicate that repotrectinib is well tolerated, exhibits both intra- and extra-cranial clinical activity and patients present partial responses, including those whose tumors harbor positive solvent front ROS1 or TRK mutations^32^. Based on the unusual binding properties of this inhibitor in the ATP binding pocket we decided to explore the therapeutic potential of repotrectinib in the context of full length ALK in a neuroblastoma setting where the gain-of-function mutations occur mostly around the α-C-helix and activation loop.

## Results

### Repotrectinib inhibits proliferation of ALK addicted neuroblastoma cells

**Figure 1:**
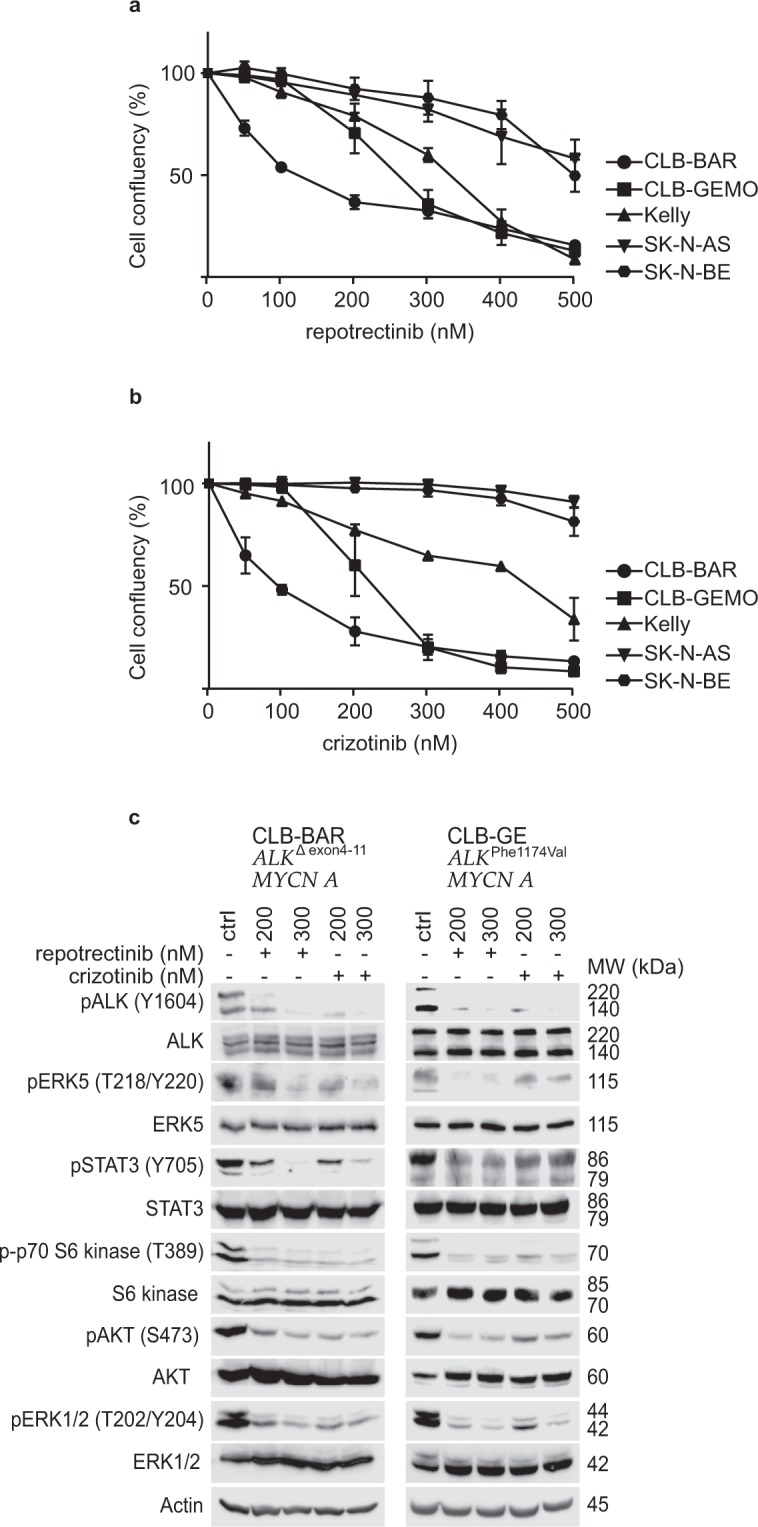
Repotrectinib inhibits proliferation of neuroblastoma cells. Neuroblastoma cells were treated with either repotrectinib or crizotinib for five days, and cell confluency monitored by live cell imaging. **(a)** Repotrectinib response curve and **(b)** crizotinib response curve. The plotted values are means ± S.D. from growth curves from three independent experiments performed in triplicate. **(c)** CLB-BAR and CLB-GE, both ALK-addicted cell lines, were treated with either 200 or 300 nM reptorectinib. Crizoitinib (200 nM and 300 nM) was employed as a positive control. Cell lysates were resolved on SDS/PAGE followed by immunoblotting for pALK (Y1604), pAKT (S473), pERK 1/2(T202/Y204), pERK5 (T218/Y220), p70 S6 kinase (T389) and pSTAT3 (Y705). Total protein for ALK, AKT, ERK 1/2, ERK5, S6 kinase and STAT3 were used as protein loading controls. Phosphorylated proteins were visualized prior to stripping and immunoblotting for total protein. CLB-BAR cells have a genomic deletion in *ALK* between exon 4–11, resulting in an ALK band of approximately 170 kDa^54^. The CLB-GE cell line expresses a mutant full-length version of ALK-F1174V, which is cleaved resulting in the detection of two bands with the antibody employed here. Blots are representative of three independent experiments.

The ALK inhibitor repotrectinib has been investigated in pre-clinical models of non-small cell lung cancer, and the results suggest an antitumor effect against cells with increased ALK activity^30–33^. In order to determine if repotrectinib has anti-carcinogenic activity in a neuroblastoma setting, we decided to study its effects on cell proliferation using two sets of neuroblastoma cell lines. The first set were ALK-addicted neuroblastoma cell lines: (i) CLB-BAR, harboring an amplified *ALK* locus with a deletion of exon 4 to 11 (Δ4-11) of *ALK* resulting in an extracellular domain ALK deletion, (ii) Kelly, which harbors an *ALK-F1174L* mutation and (iii) CLB-GE, which contains an *ALK-F1174V* mutation, which is located in the α-C-helix of the kinase domain. The second set of neuroblastoma cell lines included SK-N-AS and SK-N-BE, which are non-responsive to ALK inhibitors^34,35^. Cells were treated with increasing concentrations of either repotrectinib or crizotinib. Upon treatment with repotrectinib the proliferation rate was decreased (Fig. 1, Table 1), and the effect was more pronounced in ALK-addicted cells (almost two fold reduction in IC_50_ compared to ALK non-addicted cells) (Fig. 1a). CLB-BAR, CLB-GE and Kelly cells have an IC_50_ of 124.1 ± 4.89, 259.4 ± 6.3 and 310.9 ± 7.9 nM, respectively, while the SK-N-AS and SK-N-BE cells display higher IC_50_ values 594.8 ± 47.3 nM and 510.8 ± 16.94 nM, respectively (Table 1). Treatment with crizotinib, employed as positive control, also reduced cell proliferation of ALK addicted neuroblastoma cells as previously reported (Fig. 1b and Table 1). Immunoblotting analysis of CLB-BAR and CLB-GE cell lysates treated with either ALK TKI confirmed that repotrectinib reduced pY1604-ALK, pERK5, pSTAT3, p-p70 S6K, pAKT and pERK levels (Fig. 1c). Thus, both repotrectinib and crizotinib inhibit proliferation in ALK-addicted cell lines and inhibit phosphorylation of downstream signaling targets of ALK.

**Table 1:** IC_50_ values for repotrectinib and crizotinib in neuroblastoma cells.

Cell lines	CLB-BAR	CLB-GE	Kelly	SK-N-AS	SK-N-BE
ALK status^35^	*ALK-Δex4-11*, *ALK* amp	*ALK-F1174V*, *ALK* amp	*ALK-F1174L*, no amp	No mutation or amp	No mutation or amp
repotrectinib (nM)	124.1 ± 4.89	259.4 ± 6.3	310.9 ± 7.9	594.8 ± 47.3	510.8 ± 16.94
crizotinib (nM)	89.29 ± 4.7	221.1.9 ± 6.3	412.8 ± 18.7	796.4 ± 88.6	722.8 ± 73.25

IC_50_s were calculated at day 5 after treatment using log(inhibitor) vs normalized response. IC_50_s obtained from data in Fig. 1 are presented as mean ± S.D.

### Repotrectinib induces an apoptotic response in ALK addicted cell lines

**Figure 2:**
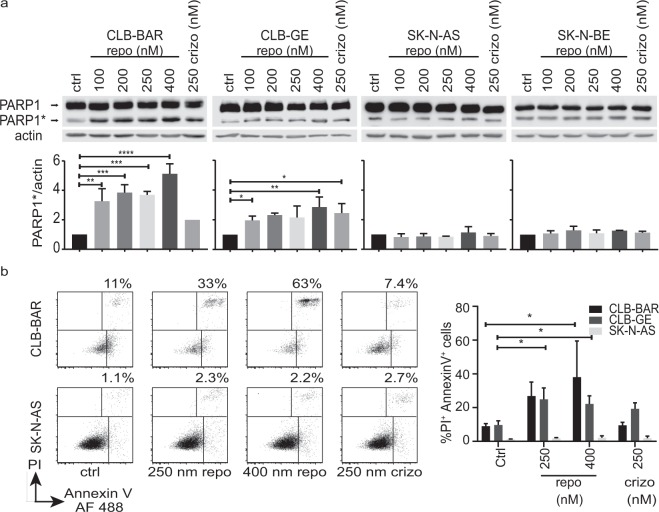
Repotrectinib treatment leads to increased PARP cleavage and apoptotic cell death in ALK-addicted neuroblastoma cells. Cells were treated for 24 h with repotrectinib (repo) at the concentrations indicated or with 250 nM crizotinib (crizo) as control. **(a)** Samples were immunoblotted with primary antibodies against either PARP1 or actin. PARP1* indicates cleaved PARP1 protein. Actin was employed as protein loading control and for normalization. Quantification represents the mean of three independent experiments +/− S.D., expressed as fold relative to non-treated controls. **(b)** Cells were harvested and stained with propidium iodide (PI) and Annexin V followed by flow cytometry analysis. A typical experiment with CLB-BAR and SK-N-AS cells showing the percentage of cells positive for both Annexin V and PI is shown at left. Quantification of the proportion of double positive cells in three independent experiments +/− S.D. using CLB-BAR, CLB-GE and SK-N-AS cells is shown at right. One way-ANOVA followed by Dunnett’s multiple comparisons test was used to determine differences between treatments: *p ≤ 0.05, **p ≤ 0.01, ***p ≤ 0.001 and ****p ≤ 0.0001.

To investigate the cellular effects of repotrectinib we first monitored caspase activity, employing PARP cleavage as readout. Cells were treated with increasing concentrations of repotrectinib (0, 100, 200, 250 and 400 nM) or 250 nM crizotinib as a positive control. Cell lysates were immunoblotted for PARP and actin as loading control. ALK-addicted CLB-BAR and CLB-GE cells displayed an increased level of cleaved PARP in response to repotrectinib treatment (Fig. 2a), with a stronger response observed in CLB-BAR cells. In contrast, the non-ALK-addicted cell lines, SK-N-AS and SK-N-BE, did not show a detectable increase in PARP cleavage. We next determined cell death by flow cytometry analysis of cells stained with Annexin V and propidium iodide (PI) (Fig. 2b and Supplementary Fig. 1). The ALK-addicted CLB-BAR and CLB-GE cell lines showed higher levels of cell death than control non-ALK-addicted SK-N-AS cells, with approximately 10% of cells positive for staining with both PI and Annexin V. Treatment with repotrectinib induced cell death in ALK-addicted cell lines, with a slightly stronger response in CLB-BAR cells. No increase in cell death was observed in the non-ALK-addicted cell line on addition of repotrectinib. Thus, treatment with repotrectinib induces an apoptotic response in ALK positive neuroblastoma cells.

### Repotrectinib inhibits phosphorylation of constitutively active ALK mutants

**Figure 3:**
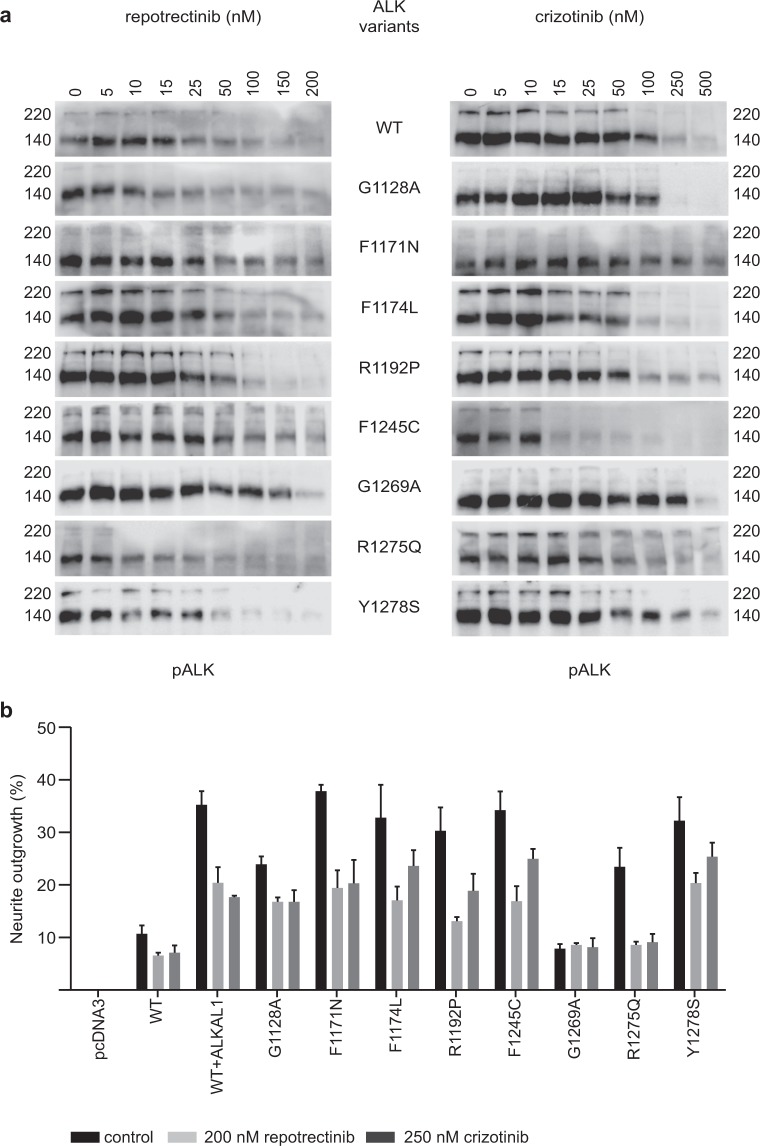
Repotrectinib inhibits ALK-Y1604 phosphorylation and neurite outgrowth driven by ALK mutants. **(a)** PC-12 cells transfected with different mutant versions of ALK were treated with either repotrectinib or crizotinib at the indicated concentrations for 4 h. Cell lysates were analyzed by immunoblotting for pALK (Y1604). Three independent experiments were performed. Representative images for the indicated ALK mutant variant are presented. **(b)** PC-12 cells were transfected with ALK mutant variants as indicated, together with GFP. Cells were treated with either 200 nM repotrectinib or 250 nM crizotinib for 48 h prior to measurement of neurite outgrowth. Cells expressing ALK-WT were stimulated with ALKAL1. GFP positive cells carrying neurites double the size of the cell body were considered positive. The data represents the percentage of GFP positive cells carrying neurites, the graph indicates the means of three independent experiments +/− S.D.

We next examined the ability of repotrectinib to abrogate the phosphorylation activity of a panel of ALK mutant variants identified in neuroblastoma cases. These included the constitutively activated ALK variants ALK-G1128A, ALK-I1171N, ALK-F1174L, ALK-R1192P, ALK-F1245C, ALK-R1275Q and ALK-Y1278S. All these ALK mutations were originally found in neuroblastoma cases and are located near the α-C-helix or the activation loop. This is different compared to resistant mutations found in NSCLC patients, which are located around the ATP-binding site of the kinase domain^23^. Wild type ALK (ALK-WT) or ALK mutants were ectopically expressed in PC-12 cells and inhibition of ALK phosphorylation by repotrectinib or crizotinib assessed by immunoblotting for pALK-Y1604. We also included analysis of the ALK-G1269A mutant, which has not been reported in neuroblastoma, but which has been described as a highly resistant mutation arising in the EML4-ALK fusion oncoprotein in NSCLC patients treated with crizotinib^25^. We observed that ALK-WT, ALK-G1128A, ALK-I1171N, ALK-F1174L, ALK-R1192P, ALK-F1245C and ALK-Y1278S showed sensitivity to repotrectinib within the range of 12–26 nM, whereas ALK-R1275Q and the EML4-ALK secondary mutation mimic ALK-G1269A required higher doses of repotrectinib to inhibit ALK-Y1604 phosphorylation (Fig. 3a, Table 2, loading controls in Supplementary Fig. 2). In general, repotrectinib inhibited the activation of constitutively active ALK variants more efficiently than crizotinib (Fig. 3a, Table 2). There was more than a four-fold difference between the ability of repotrectinib versus crizotinib to abrogate the phosphorylation of ALK-I1171N, ALK-R1192P and ALK-R1275Q.

**Table 2:** IC_50_ values of pALK-Y1604 inhibition upon treatment with either repotrectinib or crizotinib.

ALK mutant variant	crizotinib [nM]	±S.D.	repotrectinib [nM]	±S.D.	Fold change
WT	20.25	3.00	18.36	2.80	1.10
G1128A	50.31	13.07	25.87	3.90	1.94
I1171N	77.72	10.90	16.04	3.40	4.85
F1174L	13.89	1.70	12.62	1.90	1.10
R1192P	71.95	17.11	16.95	1.67	4.24
F1245C	67.16	14.80	25.56	6.10	2.63
G1269A	102.60	21.90	47.18	9.20	2.17
R1275Q	173.50	42.20	38.01	5.30	4.56
Y1278S	48.91	12.80	14.30	2.60	3.42

IC_50_s were calculated from pALK-Y1604 immunoblots normalized with actin as loading control using log (inhibitor concentration) vs normalized response (Fig. 3 and Supplementary Fig. 2). Quantification represents the mean of three independent experiments ± S.D., expressed as fold relative to crizotinib.

### Repotrectinib inhibits ALK driven neurite outgrowth in PC-12 cells

In addition to measuring ALK-Y1604 phosphorylation, we extended our analysis of repotrectinib to ALK driven neurite outgrowth. PC-12 cells are a clonal rat adrenal pheochromocytoma cell line originated from enteric cells that have the ability to differentiate and extend neurites upon protracted ERK1/2 stimulation^36^. We have previously shown that activation of ALK triggers differentiation of PC-12 cells into sympathetic-like neurons, a process that is characterized by extension of neurites^37–40^. To analyze the ability of repotrectinib to inhibit ALK driven neurite outgrowth we transfected PC-12 cells with either ALK-WT or ALK-G1128A, ALK-I1171N, ALK-F1174L, ALK-R1192P, ALK-F1245C, ALK-G1269A, ALK-R1275Q or ALK-Y1278S variants. Transfected cells were treated with either repotrectinib (200 nM) or crizotinib (250 nM) and neurite outgrowth analyzed (Fig. 3b). Inhibition of neurite outgrowth by crizotinib was in the range previously reported^38–41^. The results observed on neurite outgrowth inhibition with repotrectinib show that it has a similar ability to abrogate neurite outgrowth as crizotinib, independent of the ALK mutant variant analyzed. It should be noted that the ALK-G1269A mutant does not give rise to neurite outgrowth and therefore inhibition by either repotrectinib or crizotinib could not be assessed in this assay. Taken together, the repotrectinib inhibition profiles observed for ALK-Y1604 phosphorylation and ALK driven PC-12 cell neurite outgrowth show that this ALK TKI is able to inhibit a wide range of active ALK neuroblastoma mutants.

### Repotrectinib inhibits tumor growth in a xenograft model of neuroblastoma

**Figure 4:**
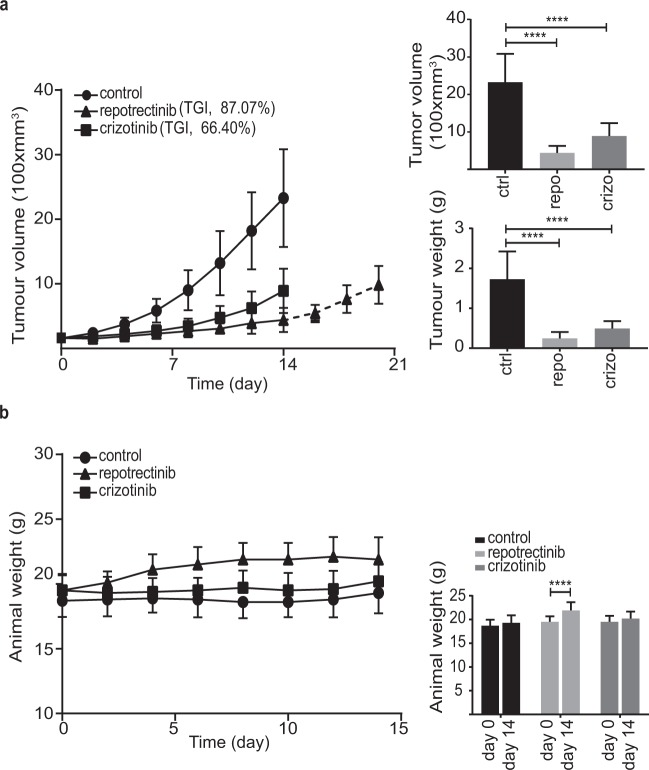
Repotrectinib inhibits tumor growth in a xenograft model of neuroblastoma. **(a)** 10 female BALB/cAnNRj-Foxn1nu mice per group were treated with repotrectinib (20 mg/kg/twice daily), crizotinib (80 mg/kg/day) or vehicle for 14 days. Five animals in the reprotectinib treated group were kept alive for a further seven days after treatment ended (dotted line, 14–21 days). Bar graphs represent mean tumor volume ± S.D. and mean tumor weight ± S.D. at day 14. TGI, tumor growth inhibition. **(b)** Animal weight was measured during the treatment for each animal from the three experimental groups. Graph indicates mean for each group at day 0 and day 14, ± S.D. One way-ANOVA followed by sidak’s multiple comparisons test was used to calculate significant differences between groups. ****Indicates *p* value ≤ 0.0001.

To further investigate repotrectinib in neuroblastoma we employed a mouse xenograft model. CLB-BAR neuroblastoma cells were injected subcutaneously and the resulting tumors treated with either repotrectinib (20 mg/kg, twice daily), crizotinib (80 mg/kg, once daily) or vehicle control. Animals treated with repotrectinib displayed minor increases in tumor volume during the 14 day treatment (Fig. 4a). Tumor growth inhibition (TGI) values of 87.07% and 66.4% were observed with repotrectinib and crizotinib, respectively (Fig. 4a). Upon repotrectinib drug release after 14 days tumor growth resumed (Fig. 4a). Tumors in the vehicle control group continued to grow reaching a significant increase compared to repotrectinib treatment after day 6 (p = 0.008) (Fig. 4a). As expected, crizotinib displayed antitumor activity in agreement with previous reports^39,40,42^. Tumor volume and weight were significantly decreased at day 14 in both repotrectinib and crizotinib groups, however, crizotinib was less effective in inhibition of tumor growth than repotrectinib (Fig. 4a). In addition to effective inhibition of tumor growth, animals treated with repotrectinib exhibited an increase in weight, showing a significant weight gain over the 14 day experiment (p < 0.0001 at day 14) (Fig. 4b).

**Figure 5:**
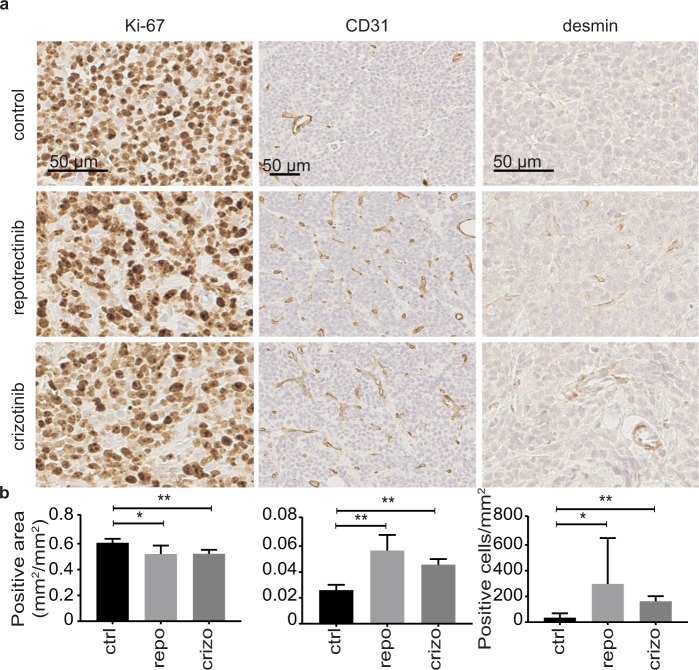
Repotrectinib treatment of neuroblastoma xenografts leads to decreased proliferation and increased vascularization. **(a)** Representative images of tumors from mice treated with either repotrectinib, crizotinib or vehicle. Tumors were stained with anti-Ki-67, anti-CD31 and anti-desmin. Images were analyzed and cropped using ImageJ. **(b)** Quantification of representative images from five tumors for each treated group, data is presented as means +/− S.D. Mann-Whitney test was used to determine significant differences between groups: *p ≤ 0.05 and **p ≤ 0.01. Scale bars indicate 50 µm.

Tumor sections from animals treated with either repotrectinib, crizotinib or vehicle were analyzed with Ki-67, CD31 and desmin as markers for proliferation and angiogenesis. Analysis of Ki-67 indicates that both repotrectinib and crizotinib led to a reduction of tumor cell proliferation (Fig. 5a,b). A significant increase in CD31 positive vessels in response to either ALK TKI shows an increased density of blood vessel penetration of tumors upon treatment compared to controls (Fig. 5a,b). To investigate the observed increase in angiogenesis further, we used desmin as a marker for pericytes. Increased desmin staining was observed in both the repotrectinib and crizotinib treated groups upon treatment with the ALK inhibitors, in agreement with the increased CD31 staining (Fig. 5a,b).

## Discussion

Despite efforts to find new targets and improve existing drugs, the standard treatments of high-risk neuroblastoma patients are currently limited to chemotherapy, local surgery, high-dose chemotherapy followed by autologous stem cell reinfusion, local radiotherapy, and maintenance treatment with retinoic compounds and immunotherapy^43^. Aberrations in *ALK* are associated with approximately 9% of all neuroblastomas including germline and sporadic cases and at an increased level in relapsed patients^6,9^. In neuroblastoma, mutations in *ALK* can induce the phosphorylation of ALK target proteins and activation of downstream signaling pathways^22^.

ALK represents a tractable target in neuroblastoma treatment and several ALK inhibitors have been developed^25^. Since the initial Children´s Oncology Group trial employing crizotinib, the first ALK TKI to be FDA approved, in pediatric ALK positive cancers^26^ several neuroblastoma patients responding to ALK TKI treatment have been reported^38,44^. Repotrectinib represents the third generation of ALK TKIs, and has a unique binding interface in the ATP-binding pocket of its target kinases^30,31^. Recent pre-clinical data in NSCLC settings and preliminary results from clinical studies show promise for the clinical applications abrogating relapse solvent front mutations occurring in fusion driven cancers^32^. Since repotrectinib shows selectivity for ALK, we decided to investigate repotrectinib in a neuroblastoma setting focusing on its effects on ALK, including ALK gain of function mutations relevant in neuroblastoma.

Here we show that repotrectinib inhibits cell proliferation in neuroblastoma cells that are dependent on ALK for growth, such as CLB-BAR, CLB-GE and Kelly cells. In neuroblastoma cell proliferation assays, the IC_50_ values observed with repotrectinib are in the same range as crizotinib, and repotrectinib is able to inhibit ALK phosphorylation over a wide range of ALK mutant variants found in neuroblastoma. Importantly, the three “hot spot” mutations found in neuroblastoma patients (ALK-F1174L, ALK-F1245C and ALK R1275Q) display low IC_50_ values for the inhibition of ALK phosphorylation with repotrectinib. Our results also show that repotrectinib induces apoptosis in ALK-addicted cell lines, resulting in increased cleavage of PARP protein as well as co-staining of Annexin V and PI indicative of late apoptotic events. The ALK-addicted cell lines used in this study exhibit high basal levels of Annexin V staining which has been observed previously in other cancer cell lines^45^ precluding quantification of early apoptotic events. Non-ALK-addicted cell lines were also sensitive at higher concentrations of repotrectinib, which may reflect the slightly broader range of targeted kinases, such as Src and Fyn^31^.

Treatment with repotrectinib as a single agent in human ALK-addicted cell line neuroblastoma xenografts resulted in robust tumor growth reduction. The effect of repotrectinib was stronger than that seen with crizotinib in xenograft experiments, despite similar IC_50_ cell proliferation values in cell culture models. We also noted that animals treated with repotrectinib gained weight during treatment, which may be due to effects on metabolism that warrant further investigation in the future.

In summary, we show that repotrectinib abrogates ALK activity in *in vitro* biochemical assays, in a manner comparable to crizotinib. However, repotrectinib is superior to crizotinib in abrogating xenograft tumor growth, likely due to its pharmacology properties, and also perhaps reflecting that repotrectinib is a potent inhibitor with a broader target kinase range. Immunostaining of the tumor material showed a significant increase in CD31 for both ALK inhibitors compared to the control group, indicating increased density of blood vessels. The increase of CD31-positive vessels could be in part due to the overall decrease in tumor volume and lead to a perceived increase of expression of CD31-positive vessels. However, we also observe an increase of the pericyte marker desmin in repotrectinib treated tumors, as shown in Fig. 5, indicating an increase in pericyte number^46^. The increase in desmin would suggest that it is not simply tumor shrinkage that leads to an increase of CD31-positive cells. Instead, the increased desmin suggests that tumor stress by ALK TKI treatment leads to hypoxia and subsequent angiogenesis and the recruitment of pericytes that bring about the overall increase of CD31-positive vessels. Increased CD31 levels have been noted before in treated tumors and have been considered to reflect changing architecture in the tumor or an endothelial cell response to the therapeutic challenge^47,48^. Altogether, these data suggest that upon treatment with the recently described ALK TKI repotrectinib, growth of ALK-driven neuroblastoma cells and xenografts are inhibited, suggesting that repotrectinib should be further explored in a neuroblastoma setting.

## Materials and Methods

### Antibodies and reagents

Primary antibodies against pAkt (S473) (#4060), pERK1/2 (Y204/T202) (#4377), pSTAT3 (Y705) (#9145), pERK5 (T218/Y220) (#3371), pALK (Y1604) (#3341), P70 S6 kinase (p85- T412/p70- T389) (#9234), β-Actin (#4970), ERK5 (#3372), AKT (#9272), STAT3 (#4904), P70 S6 kinase (#9202), PARP(#9542), CD31 (#77699), and Ki-67 (# 9027) as well as Signalstain®antibody diluent, Signalstain®Boost IHC detection reagent, Signalstain®DAB chromogent diluent and Signalstain®DAB chromogen were obtained from Cell Signaling Technology. The primary antibody for desmin (#ab32362) was purchased from Abcam and pan-ERK from BD Bioscience (#6101124). Monoclonal antibody 135 (anti-ALK) was produced in the Hallberg laboratory against the extracellular domain of ALK^49^. Horseradish peroxidase conjugated secondary antibodies goat anti-rabbit IgG and goat anti-mouse IgG, Pierce®BCA protein assay kit and Alexa Fluor 488 annexin V/dead cell apoptosis kit (cat. no. V13245) were obtained from ThermoFisher Scientific. Normal goat serum was purchased from Jackson ImmunoResearch Laboratory. pEGFPN1 vector was purchased from Clonetech, Takara Bio. Matrigel Matrix was purchased from Corning. Ingenio electroporation solution was obtained from Mirrus Bio LCC. Mayer´s hematoxylin solution, carboxymethylcellulose sodium salt and Tween-80 were bought from Sigma-Aldrich. cOmplete, EDTA-free and phosphoSTOP EASYpack were purchased from Roche Diagnostics. Repotrectinib (TPX-0005) was provided by TP Therapeutics, Inc. and crizotinib was purchased from Selleck Chemicals. ALK mutants employed in this study have been described previously^37–40^.

### Cell lines and cell culture

The neuroblastoma cells lines CLB-BAR, CLB-GE, Kelly, SK-N-AS and SK-N-BE were employed in this study. CLB-BAR and CLB-GE cell lines were obtained from The Center Leon Berard, France under MTA and were authenticated with Affymetric Cytoscan High Density array^35^. These cells were cultured on collagen coated plates. SK-N-AS, SK-N-BE and Kelly cell lines were purchased from ATCC. All cell lines were used at early passages after acquisition (less than 20). Cells were cultured in RPMI-1640 media supplemented with 10% fetal bovine serum at 37 °C and 5% CO_2_. PC-12 cells^36^ were maintained in MEM/EBSS medium supplemented with 3% FBS and 7% horse serum and a mixture of 1% penicillin/streptomycin at 37 °C and 5% CO_2_.

### Proliferation assay

Neuroblastoma cell lines were seeded into 48-well plates to achieve 30–40% confluency at the time of treatment. Repotrectinib and crizotinib were dissolved in DMSO and prepared freshly prior to addition. The concentrations of repotrectinib and crizotinib used for proliferation assays were 50, 100, 200, 300, 400 and 500 nM. The amount of DMSO did not exceed 0.1% of total medium volume. Plates were placed in an Incucyte and 16 images/well were taken every 24 h for 5 days. Each experiment was repeated three independent times and performed in triplicate. Images were taken using the 10x magnification objective for the phase contrast channel and were processed and analyzed using the Incucyte live-cell imaging system. Analysis definition was created by selecting basic analyzer, phase contrast channel and selecting 6–8 representative images. The segmentation and the minimum area (µm^2^) filters were adjusted to achieve a maximum detection of cells excluding debris. The analysis definition was done for each cell line separately and those specific parameters were used for all the images in each cell line group.

### Inhibition of ALK activity in neuroblastoma cell lines

CLB-BAR and CLB-GE cells were plated in 10 cm dishes and treated with either 200 or 300 nM of repotrectinib or crizotinib as described previously^40^. Cell lysates were collected after 1 h treatment and protein concentration was determined by BCA assay. Protein lysates were analyzed by western blotting and visualized using ECL^TM^ Prime Western Blotting Derection Reagent, Amersham^TM^ (#RPN2232) GE Healthcare. Each membrane of primary phospho-antibodies was stripped using 0.5 M NaOH for 30 min and re-blotted for total protein. β-actin was used to verify equality of sample loading. Experiments were performed in triplicates. Images were cropped using Adobe Photoshop CS6 and the final version was done using Illustrator CS6.

### Apoptosis determination

Cells were seeded in 6-well plates and treated with repotrectinib or crizotinib with the indicated concentrations for 24 h. For western blotting, cell lysates were collected using RIPA buffer (50 mM Tris-HCl pH 7.4, 1% NP40, 150 mM NaCl, 2 mM EDTA, 0.1% SDS, 1x phosphoSTOP, 1x cOmplete EDTA-free) and protein concentration was determined with the Pierce®BCA protein assay kit. Samples were immunoblotted with PARP antibody, which recognizes both full length and cleaved PARP1. Actin was used to normalize cleaved PARP1 in three independent experiments. Signal for PARP1 and actin was visualized simultaneously with immobilon Forte Western HRP substrate in an Odyssey Fc system, band intensity was determined using Image Studio Lite software. Flow cytometry was employed to analyze cells stained with Annexin V and propidium iodide as a complementary assay to PARP cleavage. Cells were collected and stained after treatment according to the manufacture’s protocol (Dead cell apoptosis kit cat. # V13241, ThermoFisher Scientific, Waltham, MA) and deposited in a 5 mL tube through cell strainer cap before analysis using an LSRII flow cytometer (BD Biosciences, San Jose, CA). Data analysis was performed using FlowJo v9.6 software (FlowJo, Ashland, OR). Image processing was done using Adobe Photoshop CS6 and Illustrator C6S.

### ALK phosphorylation IC_50_ in PC-12 cells

Cells were transiently transfected, as described previously^37,40,41^ with ALK mutant constructs or the wild type ALK construct as specified. Constructs were confirmed by sequencing. Briefly, 3 × 10^6^ cells were electroporated using 100 µL of Ingenio electroporation solution and 0.75 μg of mutant ALK constructs or 1.5 μg of the wild type variant in an Amaxa electroporator. Transfections (four) were pooled in a final volume of 10.5 mL, and 500 µL were plated per well into 24-well plates. After 48 h, cells were treated with serial dilutions of either repotrectinib or crizotinib for four hours. Cell lysates were collected and analyzed by immunoblotting. Actin, phospho-ALK-Y1604 and pan-ALK band intensity were determined using Image Studio Lite, actin was used for normalization of phospho-ALK-Y1604. pan-ALK was performed to corroborate equal loading. Images were cropped and contrast adjusted using Adobe Photoshop CS6. The IC_50_ of the ALK phosphorylation was defined as the concentration of drug that resulted in 50% levels of ALK-Y1604 phosphorylation with respect to non-treated cells.

### Neurite outgrowth assay

ALK constructs, either mutant (0.75 μg) or wild type (1.5 μg), and pEGFPN1 (0.5 μg) were co-transfected into 2 × 10^6^ PC-12 cells. After transfection, cells were diluted in 7.5 mL of medium, mixed and 300 µL were seeded into 24-well plates. The next day cells were treated with either 200 nM repotrectinib or 250 nM crizotinib, wild type ALK was stimulated with 1 µg/mL of ALKAL1^11,50^. Neurite outgrowth was analyzed 48 h post transfection^40^. Neurite formation was determined with a Zeiss Axiovert 40 CFL microscope, GFP-positive cells carrying neurites double the size of the cell body were considered positive. Experiments were performed in triplicate.

### Xenograft neuroblastoma model

In order to study the efficacy of repotrectinib we used a xenograft model of neuroblastoma. Female BALB/cAnNRj-Foxn1nu mice (Janvier Laboratory) 4–6 weeks old were housed with access to food and water ad libitum in a 12:12 light-dark cycle. The animals were allowed to acclimatize for 1 week prior to being subcutaneously injected into the left flank with 1 × 10^6^ CLB-BAR cells in serum-free medium mixed with Matrigel Matrix at a ratio of 1:1. The total injection volume was 100 µL. Once the tumor reached a volume of 150 mm^3^, mice were randomized to treatment groups using 10 animals per group. Tumor tissues treated with crizotinib or the vehicle presented in this paper have previous been used as controls in experiments by Alam *et al*.^51^. Compounds were administered orally at 80 mg/kg bodyweight daily for crizotinib and 20 mg/kg bodyweight twice daily (40 mg/kg per day) for repotrectinib for 14 days, crizotinib treatment was 4 fold higher than repotrectinib. The control group was treated with the vehicle solution; a mix of 1% carboxymethylcellulose sodium salt and 0.5% Tween-80. Tumor volume was measured by caliper every two days and calculated by the following equation: V = (p/6) × L × W2 (V, volume; p, pi; L, length; W, width). Tumor Growth Inhibition (TGI) was calculated according to: TGI = 100% x (1-((TV_t_-TV_0_)/(CV_t_-CV_0_))) where TV_t_ was the tumor volume in the treated group at the end of the experiment, TV_0_ was the tumor volume in the treated group at the beginning of the study, CV_t_ was the tumor volume in the control group at the end of the study, and CV_0_ was the tumor volume in the control group at the beginning of the treatment^32^. Animal weight was recorded every two days. All experimental procedures and protocols were performed in accordance with the Regional Animal Ethics Committee approval, Jordbruksverket (A230-2014 and 1890-2018 ).

### Tumor immunohistochemistry

Xenograft tumors were harvested after 14 days of treatment and fixed in 4% paraformaldehyde for 72 h. Fixed tumor tissue was imbedded in paraffin blocks for sectioning. 5 µm sections were obtained using a manual microtome. Heat-induced epitope retrieval (HIER) was performed using citrate buffer 0.01 M, pH 6, prior to staining. HIER was achieved by bringing the buffer to boiling, cooling for 10 sec and then sub-boiled for 5 min, repeated three times with 5 min cooling at room temperature in between. Slides were washed in distilled H_2_O before being immerged in 3% H_2_O_2_ for 15 min. After H_2_O_2_ treatment slides were washed in Tris-buffered saline-Tween 20 (TBST) for 5 min. A hydrophobic margin was then set around the samples on the slides using a water-repelling pen. Blocking was performed with 5% normal goat serum/TBST at room temperature for one hour. The following antibodies were prepared by dilution in Signalstain® antibody diluent, anti-CD31, anti-desmin and anti-Ki-67. Slides were covered with antibody diluent and placed at 4 °C for 48 h. Following washing in TBST, the slides were covered with Signalstain® Boost IHC detection reagent in room temperature for 30 min and then washed with TBST. One drop of Signalstain® DAB chromogen was added to 1 mL of DAB diluent and the mixture was added to the slides for 30–120 sec depending on the antibody, at room temperature. Following additional washing in distilled H_2_O and counterstaining with Mayer´s hematoxylin solution slides were dehydrated and mounted. All washing steps were performed 3 times for 5 min each. Sections were scanned with Hamamatsu NanoZoomer-SQ Digital slide scanner (C13140-01) with a 20× (NA 0.75) objective and analyzed using Ilastik and ImageJ or visually^52^. All images were randomly blinded. 6–7 images were chosen representing each set of images (one set containing 5 images out of each tumor category) for each antibody. The chosen images were cropped once into smaller images applying ImageJ (Supplementary Fig. 3). 6–7 images were then used as a learning base for Ilastik^53^. Four different classes were predetermined and annotated on the learning images loaded into Ilastik. The four classes were: 1. Background + stroma, 2. Negative cells, 3. Positive cells and 4. Artifact. Once the different classes and subsequently their different pixels, had been labeled, features of the labeled and the surrounding pixels were determined by Ilastik. The features were then used to train a Random Forest Classifier in Ilastik creating a prediction and segmentation map of the learning images. A batch process was performed for each set of images, applying the segmentation map acquired during the training of Ilastik, to segment the different classes in the set of images. Lastly each batch from Ilastik was processed by a macro in ImageJ (Supplementary Fig. 4), to calculate the area of positive staining. The calculation took into account the pixel size acquired from NanoZoomer Digital Pathology viewer. For slides stained with anti-desmin the positive cells were calculated visually. A 1 mm^2^ section of a representative part of the tumor was chosen using NanoZoomer Digital Pathology viewer. Positive cells within the 1 mm^2^ section were counted.

### Statistical analysis

One way-ANOVA followed by the appropriate post hoc test for multiple comparisons were used to determine differences in apoptosis, immunohistochemistry and in the xenograft experiments at significance level of 0.05. GraphPad Prism 7 was used to determine IC_50_ values. Data is presented as means of three independent experiments ± S.D.

## Supplementary information

Supplementary info
