# Supplementary material for: Repotrectinib (TPX-0005), effectively reduces growth of ALK driven neuroblastoma cells
Source: Sci Rep. 2019 Dec 18;9:19353. doi: 10.1038/s41598-019-55060-7 (PMC6920469; doi:10.1038/s41598-019-55060-7)
Supplement: Supplementary file 1 — Supplementary info [file 41598_2019_55060_MOESM1_ESM.pdf]

Repotrectinib (TPX-0005), effectively reduces growth of ALK driven neuroblastoma cells

Diana Cervantes-Madrid[1,+], Joanna Szydzik[1,+], Dan Emil Gustafsson[1], Marcus Borenäs[1], Mats Bemark[2], Jean Cui[3], Ruth Helen Palmer[1]* and Bengt Hallberg[1]*

[1]Department of Medical Biochemistry and Cell Biology, Sahlgrenska academy, University of Gothenburg, SE-405 30 Gothenburgöteborg, Sweden.

[2]Mucosal Immunobiology and Vaccine Center (MIVAC), Department of Microbiology and Immunology, Institute of Biomedicine, University of Gothenburg, SE-405 30 Gothenburg Sweden.

[3]TP Therapeutics, Inc. , 6150 Lusk Boulevard, Suite B100, San Diego, California 92121, United States.

[+]shared first authors

*Correspondence: ruth.palmer@gu.se and bengt.hallberg@gu.se; Tel.: +4631-7863815

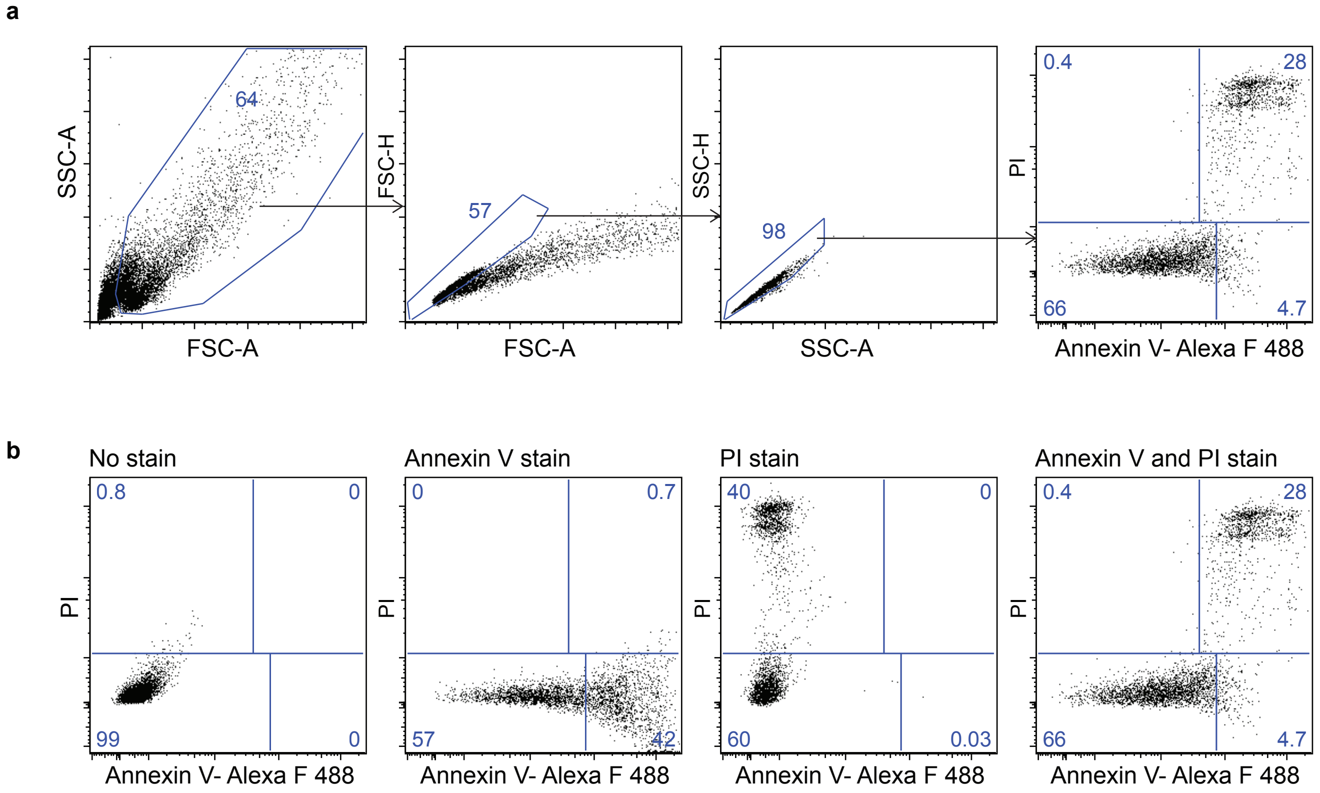

Supplementary figure 1. Gating strategy and controls for detection of Annexin V and PI. **a)** The amplification of SSC and FSC were set to include living cells, and was then gated to exclude debris. Large events were invariably doublets or larger cell clumps, and only single cells (as judged by height vs area plots) were included in the final analysis. If no gates were used before Annexin V and PI analysis, the background frequency increased but not the relative induction of apoptosis. (B) All samples were analyzed unstained (left) and fully stained (right) to ensure that autofluorescence did not influence the analysis, and in each experiment the sample treated with 400 nM repotrectinib was also stained with Annexin V or PI alone. All the cell lines showed extensive background staining with Annexin V which made it hard to set correct gates between Annexin V- and Annexin V + that did not express PI, and for this reason only the proportion of cells that were double positive were analyzed.

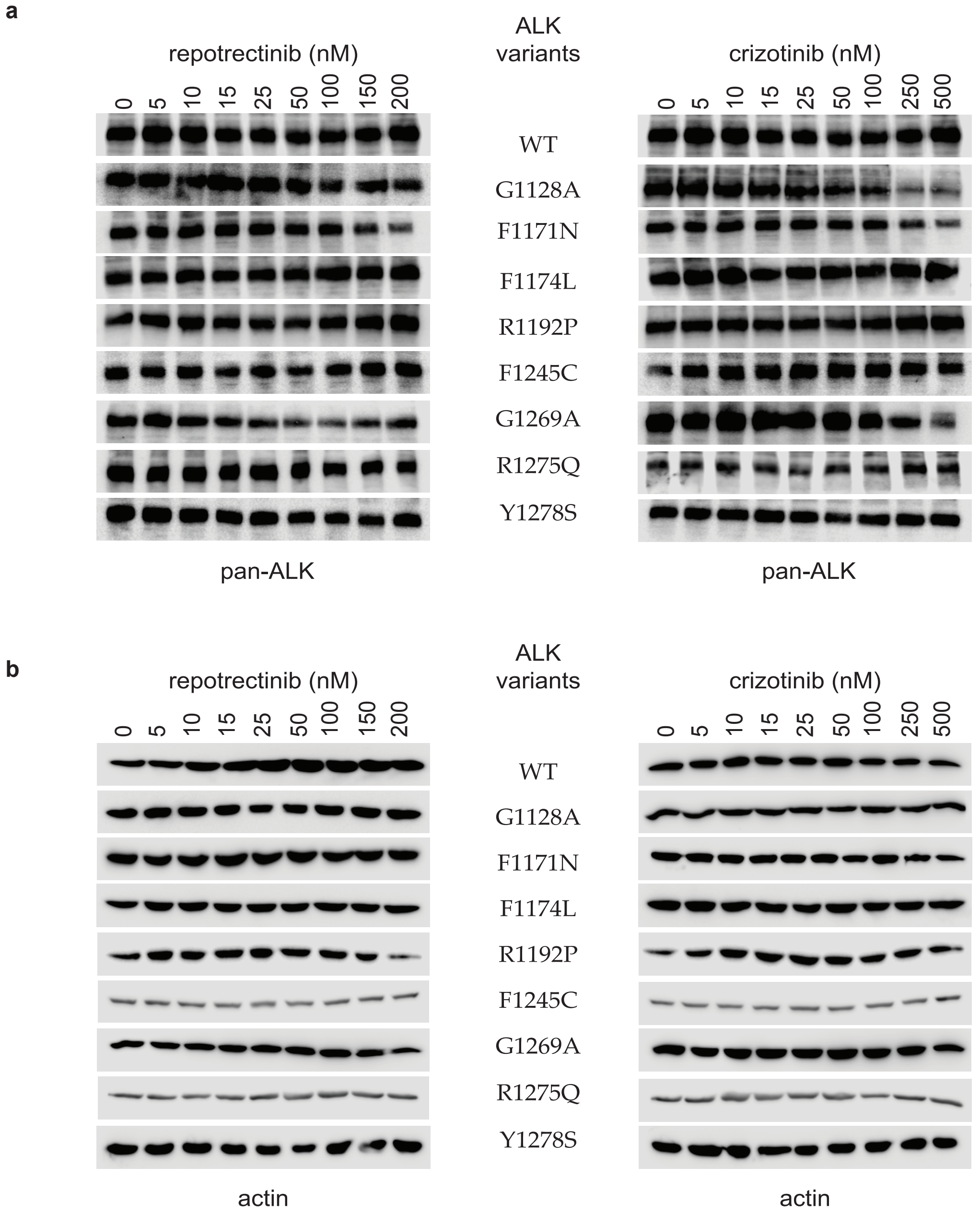

Supplementary figure 2. a) pan-ALK and b) actin corresponding to western blots showed in figure 3.

Image cropping

```
// Crop jpg image according to black square
// CCI - Hendrik Deschout - May 2018

// Get image title and rename if necessary
title = getTitle();
if (endsWith(title, ".tif")) {
	dot_index = indexOf(title, ".tif");
	title = substring (title, 0, dot_index);
	rename(title);
}

// Duplicate image
run("Duplicate...", " ");
title_duplicate = getTitle();

// Convert duplicate to 8-bit and threshold
run("8-bit");
setThreshold(50, 255);
setOption("BlackBackground", true);

// Convert thresholded duplicate to binary and apply binary operations
run("Convert to Mask");
run("Invert");
run("Fill Holes");
erode_repeats = 10;
for (i = 1; i <= erode_repeats; i++) {
	run("Erode");
}

// Determine the ROI of the square region and add to ROI manager
run("Set Measurements...", "bounding limit redirect=None decimal=3");
run("Analyze Particles...", "size=1000-Infinity add");

// Crop original image using ROI
selectWindow(title);
roiManager("Select", 0);
run("Crop");
run("Select None");

// Rename cropped image
rename(title + "_crop");

// Close other images
selectWindow(title_duplicate);
run("Close");
selectWindow("ROI Manager");
run("Close");
```

Supplementary figure 3. Program code for cropping in ImageJ 1.51j8.

```
// Measure areas corresponding to different labels as identified by Ilastik
// CCI - Hendrik Deschout - May 2018

// Ask user for folder that contains Ilastik output
dir = getDirectory("Choose a Directory");

// Get a list with all files
list = getFileList(dir);

// Define arrays in which to store the measured areas
negative_list = newArray(list.length);
positive_list = newArray(list.length);
background_list = newArray(list.length);

// Ask user for ROI width in nanometer and particle diameter in pixel
Dialog.create("User Input");
Dialog.addNumber("Pixel size (nm):", 444);
Dialog.show();
pixel_size = Dialog.getNumber() / 1000;

// Set counter
counter = 0;

// Loop through each file
for (i = 0; i < list.length; i++) {

		// Check if extension is "tif"
		if (endsWith(list[i], "tif")) {

				// Construct filepath
				path = dir + list[i];

				// Open file and get name
				open(path);
				title = getTitle();

				// Set measurements
				run("Set Measurements...", "area limit redirect=None decimal=3");

				// Measure the area for the background case
				selectWindow(title);
				run("Duplicate...", " ");
				setThreshold(1, 1);
				setOption("BlackBackground", true);
				run("Convert to Mask");
				run("Clear Results");
				run("Measure");
				background_list[counter] = getResult("Area", 0) * pixel_size * pixel_size;
				close();

				// Measure the area for the negative case
				selectWindow(title);
				run("Duplicate...", " ");
				setThreshold(2, 2);
				setOption("BlackBackground", true);
				run("Convert to Mask");
				run("Clear Results");
				run("Measure");
				negative_list[counter] = getResult("Area", 0) * pixel_size * pixel_size;
				close();
```

```
			// Measure the area for the positive case
			selectWindow(title);
			run("Duplicate...", " ");
			setThreshold(3, 3);
			setOption("BlackBackground", true);
			run("Convert to Mask");
			run("Clear Results");
			run("Measure");
			positive_list[counter] = getResult("Area", 0) * pixel_size * pixel_size;
			close();

			// Close window and update counter
			close();
			counter++;

		}
}

// Clear Results table and set counter
run("Clear Results");
counter = 0;

// Loop through each file
for (i = 0; i < list.length; i++) {

		// Check if extension is "tif"
		if (endsWith(list[i], "tif")) {

			// Store the measured areas in the Results table
			setResult("file name", counter, list[i]);
			setResult("negative area (micron^2)", counter, negative_list[i]);
			setResult("positive area (micron^2)", counter, positive_list[i]);
			setResult("background area (micron^2)", counter, background_list[i]);
			setResult("positive-negative ratio", counter, positive_list[i] / negative_list[i]);
			setResult("positive-total ratio", counter, positive_list[i] / (positive_list[i] + negative_list[i] + background_list[i]));
			setResult("negative-total ratio", counter, negative_list[i] / (positive_list[i] + negative_list[i] + background_list[i]));
			setResult("background-total ratio", counter, background_list[i] / (positive_list[i] + negative_list[i] + background_list[i]));

			// Update counter
			counter++;

		}
}
```

Supplementary figure 4. Program code for processing in ImageJ 1.51j8.
